# cGMP kinase I, cardiac hypertrophy and PDE inhibition

**DOI:** 10.1186/1471-2210-11-S1-O19

**Published:** 2011-08-01

**Authors:** Enrico Patrucco, Robert Lukowski, Sergei Rybalkin, Joe Beavo, Franz Hofmann

**Affiliations:** 1 FOR923, Institut für Pharmakologie und Toxikologie, TU, München, Germany; 2 Department Pharmacology, University of Washington, Seattle, USA

## Background

The heart responds to maladaptive pro-hypertrophic stimuli by stimulating intrinsic signals that contrast and dampen the onset and development of hypertrophy. Cyclic guanosine monophosphate (cGMP) and its downstream effector cGMP kinase I (cGKI) have been suggested to be an important anti-hypertrophic signalling pathway (1). Intracellular levels of cGMP can be raised by the action of nitric oxide (NO) and natriuretic peptides (ANP, BNP), or by inhibiting cGMP-degrading phosphodiesterases (PDE).

## Results

A growing body of evidence suggests that the PDE5 specific inhibitor Sildenafil (Sil) prevents and reverses hypertrophy and chamber remodelling in the heart of mice subjected to thoracic aorta constriction (TAC) by elevating cGMP levels and cGKI activation (1). In contrast, using a mouse model that lacks cGKI expression in CM (2), we recently showed that the absence of this kinase does not alter the onset of hypertrophy induced by TAC or isoproterenol infusion (2).

Sil is believed to increase cardiac cGMP levels, although it is unclear, if its target (PDE5) is expressed in CM (2). Sil may act on other PDEs, such as PDE1C which is abundant in CMs. It is also unclear if Sil effects are mediated by other cardiac cell types, in particular by cardiofibroblast.

## Conclusion

To answer these questions, we are currently investigating whether Sil is able to prevent hormone induced cardiac hypertrophy in the absence of cGKI in CM. Preliminary results on βRes mice show that even in the case of chronic AngII infusion, lack of cGKI In CM does not alter the Induction of hypertrophic response, at least in the initial phase (7days of AngII infusion at 2mg/kg/day). Interestingly, βRes mice showed impaired cardiac function, as indicated by decreased Fractional Shortening. Sil was able to partially block the onset of cardiac hypertrophy in WT animals, but not in βRes mice, indicating a requirement of cGKI in this process. In particular, Sil was able to block the transcription of pro-fibrotic genes such as TGFβ, CTGF, Collagen I and Fibronectin.
